# In-situ training in programmable photonic frequency circuits

**DOI:** 10.1515/nanoph-2025-0125

**Published:** 2025-06-23

**Authors:** Philip Rübeling, Oleksandr V. Marchukov, Filipe F. Bellotti, Ulrich B. Hoff, Nikolaj T. Zinner, Michael Kues

**Affiliations:** Institute of Photonics (IOP), Leibniz University Hannover, Nienburger Str. 17, Hannover, Germany; Hannover Centre for Optical Technologies, Leibniz University Hannover, Nienburger Str. 17, Hannover, Germany; Cluster of Excellence PhoenixD, Leibniz University, Hannover, Germany; Kvantify Aps, DK-2100 Copenhagen, Denmark; Department of Physics and Astronomy, Aarhus University, DK-8000 Aarhus C, Denmark

**Keywords:** machine learning, photonic computing, ultrafast optics

## Abstract

Optical artificial neural networks (OANNs) leverage the advantages of photonic technologies including high processing speeds, low energy consumption, and mass production to establish a competitive and scalable platform for machine learning applications. While recent advancements have focused on harnessing spatial or temporal modes of light, the frequency domain attracts a lot of attention, with current implementations including spectral multiplexing, neural networks in nonlinear optical systems and extreme learning machines. Here, we present an experimental realization of a programmable photonic frequency circuit, realized with fiber-optical components, and implement the *in-situ* training with optical weight control of an OANN operating in the frequency domain. Input data is encoded into phases of frequency comb modes, and programmable phase and amplitude manipulations of the spectral modes enable *in-situ* training of the OANN, without employing a digital model of the device. The trained OANN achieves multiclass classification accuracies exceeding 90 %, comparable to conventional machine learning approaches. This proof-of-concept demonstrates the feasibility of a multilayer OANN in the frequency domain and can be extended to a scalable, integrated photonic platform with ultrafast weights updates, with potential applications to single-shot classification in spectroscopy.

## 1 Introduction

In recent decades, machine learning (ML) fundamentally altered the landscape of data processing offering unprecedented capabilities in computer vision [1], medical diagnostics [2] and natural language processing [3]. While the algorithmic capabilities of ML models continue to improve, recent advancements in ML are driven by the large-scale deployment of highly specialized silicon-based electronic integrated circuits [4]. For example, tensor processing units are designed to train artificial neural networks efficiently in terms of time and energy resources. Despite these technological advancements, the large-scale deployment of ML pushes existing hardware and the associated energy consumption to their limits [5]. Thus, even more special purpose processing architectures are highly sought for, to accelerate ML in the future.

Promising architectures meeting the requirements of ML are programmable photonic circuits, which harness the interference of light to perform a computation. In particular, such systems can feature trillions of floating-point operations per second [6] and potentially consume significantly less energy than comparable integrated electronic circuits [7], [8]. A variety of ML applications have been demonstrated in photonic systems, including image classification with diffractive neural networks [9], reinforcement learning with waveguide interferometers [10] and reservoir computing harnessing optical nonlinearities [11].

Recent OANN implementations apply a hybrid approach to training, where the weights of the programmable photonic circuit are being trained on a conventional computer [12], [13], [14], [15]. More specifically, most of the OANNs implemented so far are trained on a computer and only tested on the programmable photonic circuit, limiting the benefits of the photonic system to the post training phase. In addition, current implementations of OANNs, e.g. those implemented with waveguide interferometers [12], feature a static network topology with a fixed number of input, hidden and output nodes.

The implementations of OANNs using the frequency domain for data encoding and processing has been realized before, specifically in nonlinear optical systems, using Kerr nonlinearity for nonlinear activation, see e.g. Refs. [6], [16], [17], [18], [19], [20], [21], [22], [23], [24], [25] and others. Here, we propose to implement OANNs in the frequency domain using linear optical elements with *in-situ* training, which is allowed by the optical updates of the network weights, without digital processing between layers. The data encoding in the optical frequency modes allows extensions of this network to be used for all-optical spectral data classification, e.g. spectroscopy and sensing.

A closely related approach has been previously used in the fiber-based extreme learning machines (ELMs) [23], [24], [25], [26], [27], [28], feedforward neural networks proposed in Ref. [29] and can be traced back to the original works by Rosenblatt [30]. In such neural networks, the training is implemented only in the output layer and the internal (hidden) layers are left untrained and may be assigned randomly. One of the properties of the ELMs is gradient-free training that makes them attractive for realizations in optical systems.

Here we also implement a feedforward neural network algorithm, however we employ and train multiple (here two but potentially more) optically-connected layers that allow for access to more trainable parameters.

## 2 Frequency domain processing

### 2.1 Programmable photonic frequency circuits

**Figure 1:**
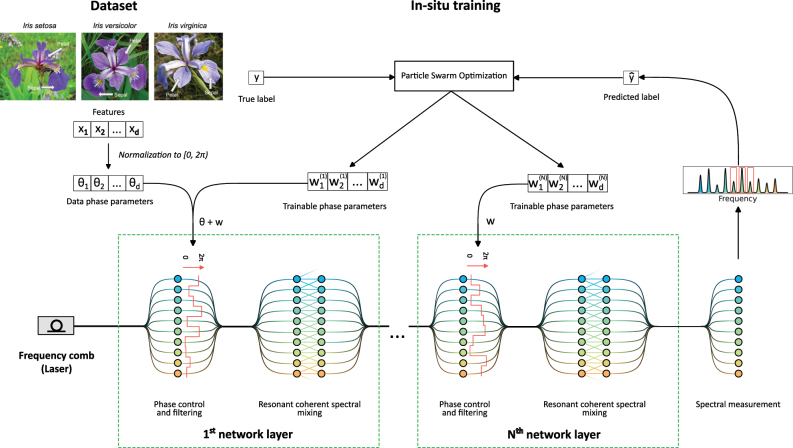
Schematic representation of a programmable photonic frequency circuit. The input features {*x*_1_, …, *x*_*d*_} are sampled from the dataset, rescaled, encoded into the phases of the frequency modes (here a frequency comb) {Θ_1_, …, Θ_2_} and subsequently processed by layers. Each layer comprises a control element to access the amplitudes and phases of the frequency modes (e.g. a programmable filter) and a mixing element to implement coherent spectral superpositions and amplitude/phase control (e.g. an electro-optic phase modulator). The weights $\left\{w_{1}^{\left(\ell \right)},\ldots ,w_{d}^{\left(\ell \right)}\right\}$ are applied to the phases of the frequency mode at each layer *ℓ*. The output, i.e. the frequency modes intensities, is recorded using a spectrometer and is interpreted as predicted classification label, using majority rule. The weights optimization is performed using particle swarm optimization of the cost function that depends on the predicted and true labels.

A programmable photonic frequency circuit is an interferometric device tailored to process information encoded in the amplitudes and phases of the frequency modes of light, see Figure 1. Such a device utilizes control elements to address amplitude and phase of individual frequency modes and mixing elements to superimpose frequency modes. By sequentially applying control and mixing elements, combined into a processing layer, our programmable photonic frequency circuit can be used to implement an OANN where the trainable weights of the network are represented by the parameters of the control elements. This approach parallels programmable waveguide circuits, where an input state is encoded into spatial modes and processed with an interferometer composed of a mesh of beam splitters and phase shifters. However, in contrast to other programmable photonic circuits, it allows for a flexible reconfiguration of the network topology. For example, the frequency mixing elements enable nearest-neighbor or next-nearest-neighbor coherent coupling of the frequency modes, which is difficult to implement in planar waveguide circuits. Moreover, because broad frequency modes intrinsically support high repetition rates, this device can be operated at high clock speeds of more than 10 GHz, surpassing electronic processing architectures.

To realize a programmable photonic frequency circuit, a variety of photonic components are available including mode locked lasers and microcombs [31], [32] to generate thousands of frequency modes, electro-optic phase modulators [33] and Bragg-scattering four-wave mixing [34] as mixing elements supporting coupling of modes over a spectral range of more than 1 THz as well as programmable filters and arrayed waveguide gratings [35] as programmable control elements.

The circuit realizes a feedforward learning algorithm. The input features {*x*_1_, …, *x*_*d*_}, where *d* is the number of features, are encoded into the phases {Θ_1_, …, Θ_*d*_} of the electromagnetic modes of the frequency comb, $E_{n}^{\left(0\right)}=|E_{n}^{\left(0\right)}|{\text{e}}^{\text{i}\Theta_{n}}$ after proper rescaling to the [0, 2*π*) interval.

In this work, we focus on the implementation with programmable filters as control elements and electro-optic phase modulators (EOPMs) as mixing elements. In our case, we apply a single-tone RF voltage *V* cos(Ω*t*) to the EOPMs, where *V* is the RF amplitude and Ω is the RF frequency. This results in the (*ℓ* + 1)-th layer *n*-th frequency mode, given by

(1) $$E_{n}^{\left(\ell +1\right)}=\underset{m}{\sum }J_{m-n}\left(v\right){\text{e}}^{\text{i}w_{m}^{\left(\ell +1\right)}}E_{m}^{\left(\ell \right)},$$

where *J*_*m*_(*v*) are the Bessel functions of the first kind and of order *m*, *v* = *πV*/*V*_*π*_ and *V*_*π*_ is the half-wave voltage of the modulator [36], [37]. The phases $w_{i}^{\left(\ell \right)}$ are the trainable parameters in the *ℓ*-th layer acquired from the programmable filter. Each layer introduces new weights $w_{i}^{\left(\ell +1\right)}$ that contribute linearly to the phase of the field modes. The weights are rescaled to fit on the interval [0, *π*], in order to not limit the redundancy due to the periodicity of the trigonometric functions. However, the mixing involves all of the field modes and leads to complex interference patterns that allow us to train the circuit. The number of the frequency modes utilized during the training equals the number of the input features with the additional modes, that result from the mixing, being extinguished by the programmable filter in the layer.

Explicitly, the action of the programmable photonic frequency circuit layer by layer can be written as

(2) $$E_{n}^{\left(1\right)}=\underset{m}{\sum }J_{m-n}\left(v\right)E_{m}^{\left(0\right)}{\text{e}}^{\text{i}w_{m}^{\left(1\right)}},$$

(3) $$E_{n}^{\left(2\right)}=\underset{m}{\sum }J_{m-n}\left(v\right)E_{m}^{\left(1\right)}{\text{e}}^{\text{i}w_{m}^{\left(2\right)}}.$$

The output is given by the modes intensities

(4) $$I_{n}=|E_{n}^{\left(N\right)}{|}^{2}.$$

The number or the intensities used equals the number of classes as described in Section 3.

### 2.2 Experimental implementation

**Figure 2:**
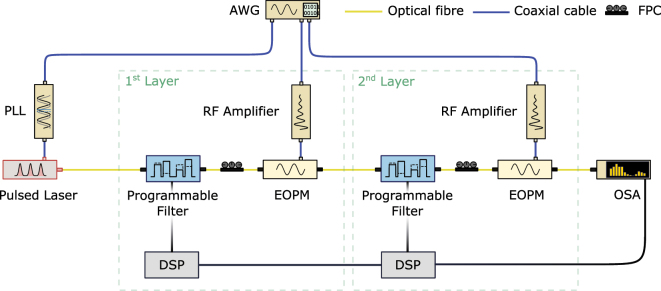
Schematic representation of the fiber-based setup implementing a programmable photonic frequency circuit with 2 sequential processing layers. The processing layers are seeded by a pulsed laser emitting a train of pulses with <45 ps duration at a repetition rate of 50 MHz. Each processing layer comprises a programmable filter to upload data and/or trainable weights interfaced by a digital signal processing (DSP) and an electro-optic phase modulator to superimpose frequency modes. The spectrum is read out with an optical spectrum analyzer (OSA) and propagated to a digital computer to compute the feedback. Further components: arbitrary waveform generator (AWG), phase-locked loop (PLL), fiber polarization controller (FPC).

In our experiment, we implemented a programmable photonic frequency circuit with two layers in a fiber-based setup, as shown in Figure 2. Both processing layers consisted of a programmable phase and amplitude spectral filter (Waveshaper WS 4000A, Coherent), implementing the control element, followed by an electro-optic phase modulator (EOPM, EO Space), implementing the mixing element. The optical inputs were four frequency modes with a single-mode bandwidth of 9.71 GHz (FWHM) and a spectral separation of 34 GHz, filtered from the spectrum of a mode locked laser (MLL, Menlo C-Fiber 780). To maintain coherence throughout the device, the MLL repetition rate was synchronized with the phase modulators in the subsequent processing layers. This synchronization is achieved with a phase-locked loop (PLL), locked to the repetition rate of an external 1 GHz reference provided by an arbitrary waveform generator (AWG, Keysight M8194A).

**Figure 3:**
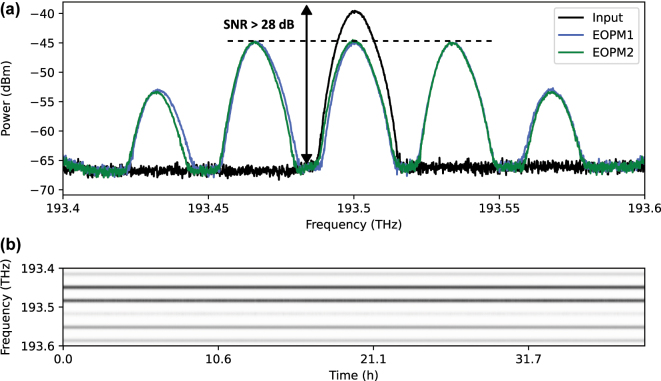
Characterization of the experimental setup. (a) Sideband spectra for the two EOPMs, calibrated to the power equalization of zeroth and first sideband (dashed line). The signal to noise ratio (SNR) of 28 dB was limited by the intrinsic noise floor of the spectrometer that was used. The mode spacing was 34 GHz in this calibration measurement and the total spectral power was 24.1 μW. (b) Spectral intensities at the output of the circuit for a given random phase configuration. An uploaded phase profile in the two layer system keeps constant for more than 32 h proving the long-term stability of the system.

The broadband emission of the MLL contains optical sidebands outside the support (i.e. the set of original modes), which can couple to the four frequency modes in the subsequent layers. Additionally, phase modulation in each layer generates optical sidebands outside the support. To suppress these undesired sidebands, the incoming light in each layer is spectrally filtered by the programmable filter before spectral phases are applied to encode the trainable parameters and upload the data to the frequency circuit. To superimpose the frequency modes in each layer, the EOPMs were driven in resonance to the input modes at 34 GHz. Each of the frequency modes had a spectral width (FWHM) of 9.7 GHz and the average total input power was 144.2 μW combined for the 4 mode input system. In the two layer configuration the optical loss of the setup was 15.6 dB. The RF driving power was adjusted to equalize the zeroth and first optical sidebands, enabling balanced superpositions between adjacent modes, see Figure 3a. This choice of the RF driving power is convenient. However, its value can be treated as a hyperparameter of the circuit. After passing through the layers, the optical output was measured using an optical spectrum analyzer (WaveAnalyzer 1500A, Coherent Inc.) with a spectral resolution bandwidth of 150 MHz over a range of 200 GHz. The optical connections between the components were established using non-polarization-maintaining single-mode fiber with the total length of the fibers being less than 10 m. With the relatively low power used in the experiment, nonlinear effects associated with the Kerr-nonlinearity do not play a significant role in our experiment.

## 3 Results

### 3.1 Dataset and pre-processing

To demonstrate the ML capabilities of our device, we performed *in-situ* training and testing of multiclass classification on an artificial dataset, which can be thought of as an artificial extension of the Iris dataset [38] with the fiber-based setup described in Section 2.2. The Iris dataset is a common dataset containing 150 samples of flowers each characterized by 4 features (sepal length, sepal width, petal length, petal width) and labeled as 3 classes (setosa, versicolor and virginica). The classification of this dataset was used to benchmark ML in photonic systems before [39], [40].

The Iris dataset properties, specifically the number of features and classes, fit well to the current configuration of our proof-of-concept setup. It contains, however, only 150 samples and thus we chose to use an artificial dataset with a much larger sample size for training and testing. While many other standard datasets are available for training and testing classification algorithms, see, e.g. MNIST [41], they usually employ many more features in comparison to the Iris dataset and are not feasible in the current proof-of-concept setup. However, we expect a scaled version of the presented approach to be able to tackle such larger problems.

To construct the dataset, a principal component analysis (PCA) was performed. The statistical distributions of the samples of each class were fitted by Gaussian distributions using the mean and variance of the principal components. For each class, 5,000 artificial data points were sampled from the Gaussian distributions and the inverse PCA was applied to generate a total of 15,000 samples in the original feature space for all three classes. This dataset was further divided into 12,000 samples for training (4,000 for each class) and 3,000 samples for testing (1,000 for each class). While the dataset is new and artificial, we refer to its origins from the Iris dataset by using the same names for the features and classes. The samples were physically uploaded into the phase profile of the programmable filter in the first layer of the circuit. Note, that we do not use the spectral amplitudes for the encoding of the input data. This dataset is available online and can be accessed via [42].

### 3.2 Training results

For the experimental multiclass classification, we performed a total of 20 repetitions (epochs) of the experiment, with the weights initialized randomly. In each epoch, 25 iterations were performed to optimize the circuit’s weights via particle swarm optimization (PSO) [43]. In contrast to gradient-based optimizers, struggling with local minima, the PSO algorithm is a gradient-free global optimizer suited for high-dimensional optimization problems. In order to optimize for the hyperparameters of the OANN and the PSO algorithm, we simulate the training of the programmable photonic frequency circuit numerically. The electromagnetic modes of the frequency comb are treated as a superposition of well-separated Gaussians in frequency space and the processing layers operation are modeled as linear multiplicative transformations [37]. We find that a swarm size of 16 particles is reasonable for the PSO algorithm to converge.

**Figure 4:**
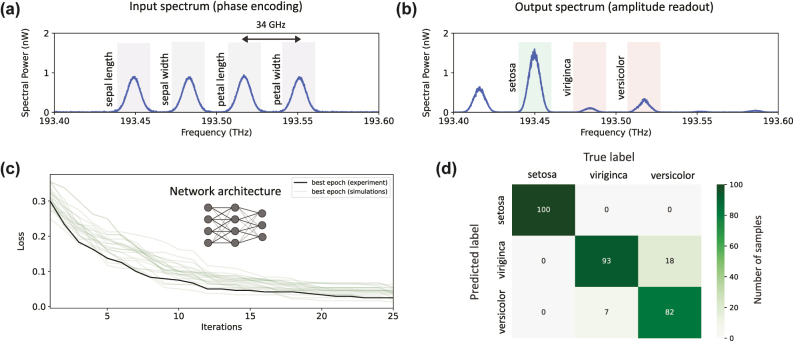
Experimental results of the multiclass classification for the artificial extension of the Iris dataset with a (4, 4, 3) network architecture. (a) Features of a sample are phase encoded into four frequency modes generated from the mode locked laser. (b) To infer the classification result, the output amplitudes are read out. Each output bin labels a different class. In the example provided here, a sample belonging to setosa was encoded and processed by the programmable photonic frequency circuit. The output spectrum reveals most intensity at the bin labeled setosa, indicating a correct classification for this sample. (c) Training progress of the *in-situ* training (black) for 10 epochs each with 25 iterations. As a reference and baseline, 20 runs on a numerical simulation (green) are shown. (d) Confusion matrix of the best epoch evaluated over the test set with 100 samples in each class (300 samples total). The total classification accuracy is 91.7 %.

To classify samples from the dataset, three of the frequency modes at the circuit output were assigned to the class labels (setosa: 193.449 THz, virginica: 193.483 THz, versicolor: 193.517 THz), see Figure 4b. Following the uploading and processing of a sample, the predicted class was determined by the output frequency mode with the highest optical intensity.

To update the weights of the circuit, in each iteration a batch of 12 samples (four from each class) uniformly sampled from the training dataset was classified and the portion of incorrectly classified samples from this batch was calculated. This batch accuracy was used as the loss function for the weights update in the next iteration. While categorical cross entropy (CCE) is frequently used as a loss function for multi-class classification, in our case the batch accuracy yields better training results.

To determine the classification accuracy after an epoch of 25 iterations, we classified 300 samples uniformly chosen from the test dataset (100 from each class). The overall runtime of one epoch of the *in-situ* training is given by *T* = *N*(iterations = 25) ⋅ *N*(particles = 16) ⋅ *N*(batchsize = 12) ⋅ Δ*t*, where Δ*t* is the time it takes to upload and evaluate a single sample from the dataset. In our experiment, this time was severely limited by the high latency of the programmable filter (1.1 s), resulting in an overall training time of approximately 5,300 s per epoch. Harnessing electro-optical components for spectral phase manipulation, we estimate that this latency can be reduced to <1 µs, which in our case would give rise to an overall training time of a few seconds. In this case, the training time would no longer be limited by the optical circuit, but by the classical optimizer that is run on the computer.

Training results for the multiclass classification on the extended Iris dataset are shown in Figure 4. We achieved a classification accuracy of 91.7 % on the test set after 25 iterations proving the capabilities of our experiment. In order to compare the programmable photonic frequency circuit performance with a conventional neural network, we implemented a deep neural network with one linear and two nonlinear hidden layers using pytorch [44] and achieved 95.8 % accuracy on the same dataset. A linear classifier, i.e. one not employing nonlinear activation between layers, showed similar accuracy of 95.6 %. The discrepancy in training accuracy between the conventional neural networks and the experimental realization could be explained by the extperimental noise. This is supported by our numerical simulations of the experiment that show a closer accuracy of 94.8 %. Note, that a perfect distinction between virginica and versicolor is not possible due to the overlap of data which is reflected in the confusion matrix in Figure 4d.

The stability of the system was critical to ensure consistent results, as *in-situ* training for 20 epochs required over 24 h in the fiber-based experiment. Extrinsic disturbances, such as phase drifts caused by thermal length fluctuations of the optical fibers, can potentially alter the circuit’s weights, compromising performance. We checked the stability of the system experimentally by observing the spectral intensities at the output for more than 32 h. As can be seen in Figure 3b the uploaded phase profile is maintained for the whole duration. A trained classification model is expected to be persistent, allowing to reliably re-uploaded weights and data into the system reproducing the same results. To check persistence for our implementation, we performed classification on the same test set after training again, achieving comparable classification accuracy of 90.3 %, indicating that the system was stable.

One of the major advantages of our system is its versatility and the ability to be dynamically reconfigured to better fit the task at hand. Namely, the same experimental setup can be used for classification of dataset with different number of features and classes, using various number of weights and, in principle, processing layers. Importantly, this scalability is not easy to achieve in other OANNs realizations, e.g. reconfiguration of a diffractive deep neural network would require reprinting of the passive diffractive layers. Using the programmable filter that covers the entire telecommunication C-Band, with 5 THz of optical bandwidth and our operating bandwidth of 34 GHz, allows us to fit approximately 140 frequency channels i.e. network nodes. A caveat, however, is the limited coupling range of the EOPM for the parameters used in this work. It could be addressed by increasing the voltage applied to the EOPMs and/or cascading more layers of the networks. Another approach to scalability is to use a photonic chip-integrated platform that would decrease the system size and improve the system’s processing speed.

To prove that the network topology of our device is versatile and can be dynamically reconfigured, we performed another classification on the Palmer penguin’s dataset [45], which we extended in the analogous to the Iris dataset fashion. However, we kept only one categorical data feature of the original dataset before using it for the extension, in order to better fit over experimental parameters. The resulting dataset has five input features per sample and requires a different network topology with five frequency input modes to encode features into the phases. With the two layers fiber-based programmable photonic frequency circuit setup we achieved a classification accuracy of 86.8 %. The classification of the dataset, using a conventional linear classifier and a deep neural network with one linear and two nonlinear hidden layers, were both performed with approximate accuracy of 97.4 %. Note that the numerical simulations of the experiment show closer accuracy of 94.3 %, which again supports that the source of the discrepancy is the experimental noise. This dataset is also publicly available at Ref. [46].

## 4 Discussion

In summary, we proposed a programmable photonic frequency circuit to implement OANNs in the frequency domain and experimentally demonstrated in a fiber-based experiment their capabilities towards multiclass classification of labeled data by training on the artificial extension of the Iris dataset. In contrast to previous implementations, which are typically trained on a digital computer and only tested on photonic hardware, we performed *in-situ* training using several optically-connected layers, the first, to the best of our knowledge, in a setup that exploits the frequency domain, highlighting the opportunities and challenges of this approach. Moreover, this device holds the potential for fully chip-integrated coherent processing by harnessing recent developments in the generation of microcombs, manipulated using arrayed waveguide gratings and spectrometers. In addition, the circuit operates with signals and data encodings that are frequently used in telecommunications, providing a mature infrastructure to deploy these systems.
